# Response of Transgenic Potato Plants Expressing Heterologous Genes of ∆9- or ∆12-Acyl-lipid Desaturases to *Phytophthora infestans* Infection

**DOI:** 10.3390/plants11030288

**Published:** 2022-01-21

**Authors:** Elena V. Tsypurskaya, Tatiana N. Nikolaeva, Petr V. Lapshin, Tatiana L. Nechaeva, Natalya O. Yuorieva, Ekaterina N. Baranova, Marina K. Derevyagina, Lyudmila V. Nazarenko, Irina V. Goldenkova-Pavlova, Natalia V. Zagoskina

**Affiliations:** 1 K.A. Timiryazev Institute of Plant Physiology, Russian Academy of Sciences, 127276 Moscow, Russia; niktat2011@mail.ru (T.N.N.); p.lapshin@mail.ru (P.V.L.); nechavtatyana.07@yandex.ru (T.L.N.); yuorieva@mail.ru (N.O.Y.); irengold58@gmail.com (I.V.G.-P.); 2 N.V. Tsitsin Main Botanical Garden of Russian Academy of Sciences, Botanicheskaya 4, 127276 Moscow, Russia; 3 All Russia Research Institute of Agricultural Biotechnology, Russian Academy of Agricultural Sciences, 127550 Moscow, Russia; 4 Russian Potato Research Center, Lorkha 23, 140052 Kraskovo, Russia; marina.1958derevyagina@mail.ru; 5 Department of Biology and Human Physiology, Institute of Natural Sciences and Sports Technologies, Moscow City Teachers’ Training University, 129226 Moscow, Russia; nlv.mgpu@mail.ru

**Keywords:** *Solanum tuberosum* L., transgenic plants, acyl-lipid desaturases, resistance to phytopathogen, *Phytophthora infestans*, lipid peroxidation, phenolic compounds, flavonoids

## Abstract

Late blight is one of the most economically important diseases affecting potato and causing a significant loss in yield. The development of transgenic potato plants with enhanced resistance to infection by *Phytophthora infestans* may represent a possible approach to solving this issue. A comparative study of the leaf response in control potato plants (*S.tuberosum* L. cultivar Skoroplodnyi), control transgenic plants expressing the reporter gene of thermostable lichenase (transgenic *licB*M3 line) and transgenic plants expressing cyanobacterial hybrid genes ∆9-acyl-lipid desaturase (transgenic *desC* lines) and ∆12-acyl-lipid desaturase (transgenic *desA* lines) to infection with *P. infestans* has been performed. The expression of desaturase genes in potato plants enhanced their tolerance to potato late blight agents as compared with the control. The lipid peroxidation level raised in the leaves of the control and transgenic *desA* plants on third day after inoculation with *P. infestans* zoospores and remained the same in the transgenic *desC* plants. The number of total phenolic compounds was increased as early as on the second day after infection in all studied variants and continued to remain the same, except for transgenic *desC* plants. Accumulation of flavonoids, the main components of the potato leaf phenolic complex, raised on the second day in all studied variants, remained unchanged on the third day in the control plants and decreased in most transgenic plants expressing desaturase genes. The results obtained in our study demonstrate that the expression of genes of Δ9- and Δ12-acyl-lipid desaturases in potato plants enhanced their resistance to *P. infestans* as compared with the control non-transgenic plants due to concomitant accumulation of phenolic compounds, including flavonoids, in the leaves. All these changes were more pronounced in transgenic *desC* plants, which indicates that the Δ9-acyllipid desaturase gene appears to be a potential inducer of the production of biological antioxidants in plant cells.

## 1. Introduction

An important feature for the future of plants is their ability to adapt to environmental conditions and the effects of various abiotic and biotic factors, including infection with pathogens [1]. The adaptation is accompanied by corresponding changes in their metabolism, including those caused by an increase in the number of reactive oxygen species (ROS) [2,3,4]. The antioxidant system, comprising mani-fold high- and low-molecular-weight compounds, is involved in the maintenance of ROS balance [5,6]. Low-molecular-weight antioxidants include phenolic compounds, which are among the most abundant secondary metabolites of plants [7,8]. Due to their chemical structure, they are able to interact with ROS, thereby protecting cells from their toxic effect [9,10]. 

Phenolic compounds are known to be involved in the protection of plants from many stress impacts, including phytopathogens [11,12]. They prevent the penetration of plant tissues by pathogenic microorganisms and inhibit their growth and development as well as the formation of cell-toxic metabolites [13,14]. For example, quercetin and its derivatives have been shown to inhibit the growth of the fungus *Neurospora crassa* on *Arabidopsis thaliana* [15].

Transgenic plants expressing heterologous genes can be used to research both the regulation of complex metabolic pathways and the response of plants to environmental factors [16,17]. This approach was applied to assess the impact of the introduced heterogeneous desaturase genes with different substrate specificities on the defense response of potato plants to phytopathogen infection [18]. Desaturases introduce double bonds (C=C) to chains of fatty acids and display specificity towards the length of a hydrocarbon chain and the site where it is formed [19]. In particular, ∆9-acyl-lipid desaturase introduces the first double bond, converting stearic acid (18:0) to oleic acid (18:1), while ∆12-acyl-lipid desaturase is involved in the creation of the second double bond in oleic acid (18:1), which leads to its conversion to linoleic acid (18:2). These enzyme activities determine physical properties of biological membranes in plant cells, namely, the level of unsaturation of fatty acids [18,20].

The transgenic plants that express the genes encoding heterologous cyanobacterial desaturases have attracted the attention of researchers in studies of their contribution to fatty acids’ unsaturation index as well as resistance to various abiotic and biotic environmental factors [18,21]. It has been shown that the tobacco plants expressing ∆9-acyl-lipid desaturase encoding the gene of *Synechococcus vulcanus* showed an increased proportion of polyunsaturated fatty acids in their membrane lipids, altered ultrastructure of chloroplasts and increased tolerance to hypothermia [22]. The resistance to the pathogen *Colletotrichum gloeosporioides* in transgenic avocado plants increased with the expression level of ∆9-stearoyl-ACP-desaturase [23]. Expression of the gene of ∆12-acyl-lipid desaturase of *Synechocystis* sp. PCC6803 increased the in vitro resistance of the potato cultivar Desnitsa to late blight with a lower level of lipid peroxidation (LPO) as compared with the control [24]. All these facts indirectly suggest that the function of the antioxidant system is changed in the transgenic plants expressing heterologous genes of desaturases. However, data on the accumulation of phenolic antioxidants in the transgenic plants expressing the genes coding for different desaturases, including the case of infection with phytopathogens, are almost absent.

In this work, we use control and transgenic potato plants expressing the genes of cyanobacterial ∆9- and ∆12-acyl-lipid desaturases to assess their response to infection with zoospores of *P. infestans,* a pathogenic oomycete, and attempt to clarify whether the observed resistance is determined by an altered LPO level and accumulation of total phenolic compounds and flavonoids.

## 2. Results

### 2.1. Morphophysiological Characteristics of Potato Plants

**Figure 1:**
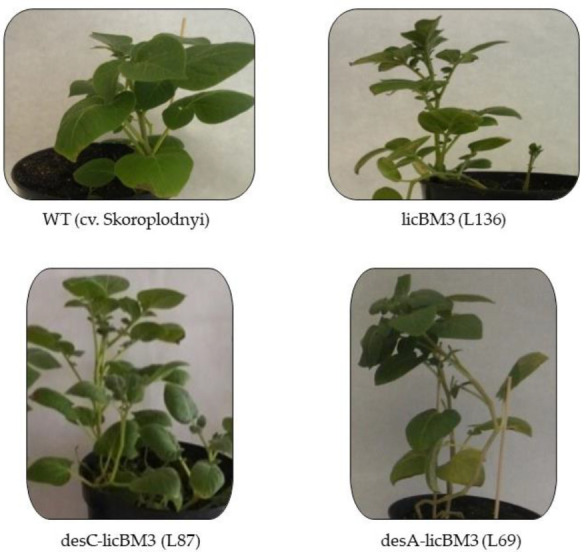
Appearance of control cultivar Skoroplodnyi and transgenic lines plants of potato, expressing the genes *licB*M3, *desC-licB*M3 or *desA-licB*M3 (plant age, 60 days). WT (cv. Skoroplodnyi)—control plants; *licB*M3 (L136)—transgenic potato plants (line 136) expressing the reporter gene of thermostable lichenase (*licB*M3); *desC-licB*M3 (L87)—transgenic potato plants (line 87) expressing the hybrid genes of ∆9-acyl-lipid desaturase from *Synechococcus vulcanus* translationally fused with the sequence of reporter gene *licB*M3; *desA-licB*M3 (L69)—transgenic potato plants (line 69) expressing the hybrid gene of ∆12-acyl-lipid desaturase from *Synechocystis* sp. PCC6803 translationally fused with the sequence of reporter gene *licB*M3.

The transgenic *desA* line of potato plants differed significantly from control plants of the cv. Skoroplodnyi in height, number of internodes and total weight, while the plants of the control transgenic *licB*M3 line and transgenic *desC* line were similar to the control (Figure 1, Table 1). 

However, another pattern was observed for the leaves of the plants. The weight of leaves was statistically equal in all plant lines studied. The control plants and plants of transgenic *licB*M3 and *desC* lines had a higher proportion of leaves (80%) to total plant biomass as compared to plants of the transgenic *desA* line (53%). Based on the results, we can assume a significantly lower rate of leaf formation in *desA* plants (Figure 1).

Determination of the water content in plant leaves, as an indicator of their physiological status, did not reveal statistical differences in the studied variants of potatoes (Table 1). However, the water content in the leaves of *desA* plants was higher, exceeding that of the control variant by an average of 15%.

**Figure 2:**
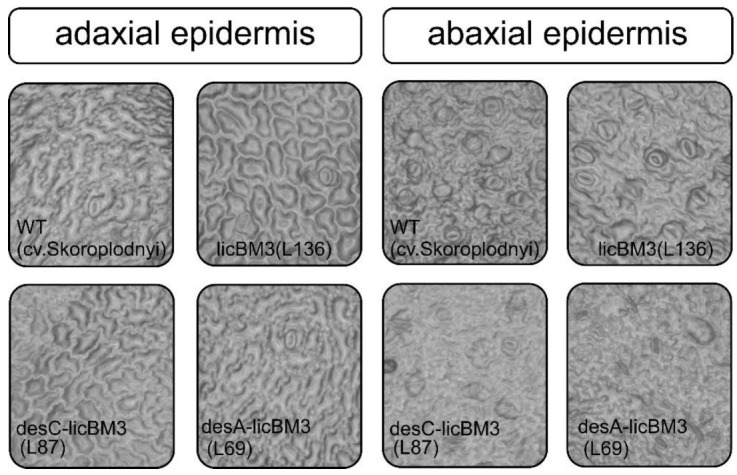
The location of stomata on the abaxial and adaxial leaf surfaces of control potato plants of the cultivar Skoroplodnyi and transgenic lines, expressing the genes *licB*M3, *desC-licB*M3 or *desA-licB*M3 (plant age, 60 days).

Stomata were observed as single formations in the epidermis of the leaf adaxial side in control and transgenic potato plants (Figure 2, Table 2).

However, the number of stomata was high, especially in the control plants, in the epidermis of the abaxial leaf side. There were no significant differences in the stomata area in any of the variants studied. Determination of the stomatal index showed its high value for the abaxial leaf surface, which was correlated with the number of stomata.

### 2.2. Inoculation of Potato Leaves with Phytophthora infestans

**Figure 3:**
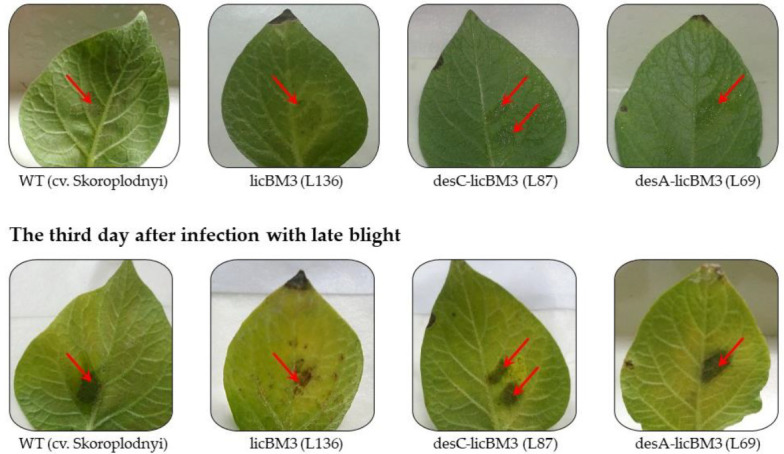
Detached leaf assay of the control and transgenic plants of potato cultivar Skoroplodnyi expressing the genes *licB*M3, *desC-licB*M3 or *desA-licB*M3 with *P. infestans* isolate 1.2.3.4.5.6.7.8.9.10.11 XYZ.

Infestation of plant leaves with *P. infestans* and the subsequent development of zoospores on them was accompanied by the formation of necrotic areas. A similar response was observed on the control and transformed leaves with the genes of cyanobacterial desaturases potato plants (Figure 3).

Symptoms of infection were observable the second day after some leaves of the control and transgenic potato plants were inoculated with the suspension of *P. infestans* zoospores, and symptoms further developed on the third day (Table 3).

The transgenic lines of *desC* and *desA* plants displayed statistically equal and reliably higher tolerance to the phytopathogen as compared with the control and the line expressing the reporter gene *licBM3*. 

Consequently, expression of the genes encoding desaturases with different substrate specificities (∆9- and ∆12-acyl-lipid desaturases) in potato plants enhanced their resistance to the infection with *P. infestans*.

### 2.3. Lipid Peroxidation in Potato Plant Leaves

**Figure 4:**
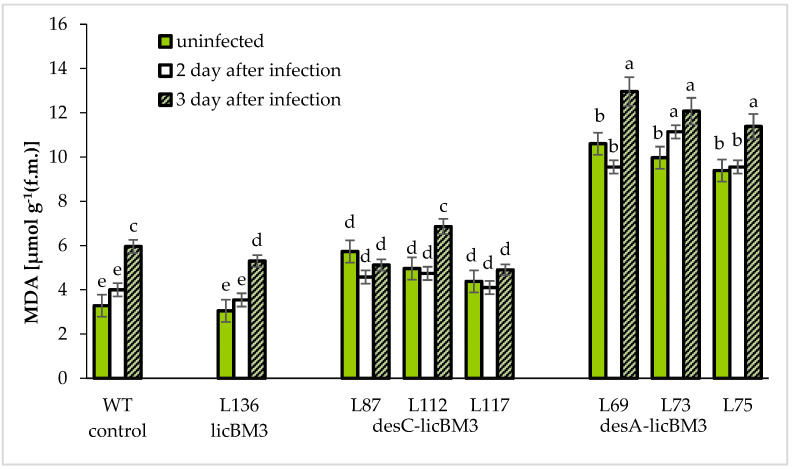
Changes in the content of malondialdehyde (MDA) in the leaves of the control plants of potato cv Skoroplodnyi and transgenic plants, expressing gene *licB*M3, gene *des*C-*licB*M3 and gene *desA*-*licB*M3 after infection with *P. infestans* isolate 1.2.3.4.5.6.7.8.9.10.11 XYZ. Means ± SDs, *n* = 3. Different Latin letters above the bars denote statistically significant differences at *p* *≤* 0.05.

It should be noted that a significantly higher LPO level in all *desA* plants, which exceeded the control by almost two-fold, was observed (Figure 4).

The infection of leaves with *P. infestans* elevated the MDA content in the control plants and the transgenic *licBM3* line on the third day of infection (by 80% as compared with the uninfected plants) (Figure 4). As for the transformants expressing the *desC-licB*M3 gene (*desC* plants), the MDA content in most cases remained the same except for line 112, in which it increased by 35% on the third day after inoculation (as compared with the uninfected plants). The MDA content in leaves of the transgenic plants expressing the *desA-licB*M3 (*desA* plants) increased after infection on the second day after inoculation in line 71 and on the third day in lines 69 and 75 (by 15% in all variants). These data suggest that expression of the *desA-licB*M3 gene in the potato plants (cv. Skoroplodnyi) causes more pronounced changes as compared with the transgenic potatoes carrying the *desC-licB*M3 gene.

### 2.4. Content of Phenolic Compounds in Potato Leaves

**Figure 5:**
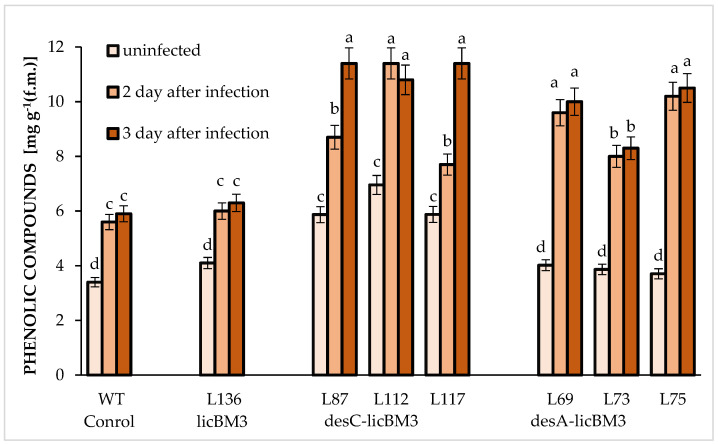
Changes in the content of total phenolic compounds in the leaves of control plants of potato cultivar Skoroplodnyi and transgenic lines, expressing gene *licB*M3, gene *desC-licB*M3 (*desC* plants) and gene *desA-licB*M3 (*desA* plants) after infection with *P. infestans* isolate 1.2.3.4.5.6.7.8.9.10.11 XYZ. Means ± SDs, *n* = 3. Different Latin letters above the bars denote statistically significant differences at *p* *≤* 0.05.

As is evident from Figure 5, the total of phenolic content increased (by 60%) in the leaves of control plants and in the leaves of transgenic plants expressing the reporter gene *licB*M3 on the second day after infection with *P. infestans* and continued to remain unchanged. A similar pattern was observed in leaves of *desA* plants. However, the accumulation of these secondary metabolites was greater in this case, amounting to over 200% as compared with the uninfected leaves. Transgenic *desC* plants displayed another pattern: the amount of total phenolic compounds in the leaves of lines 87 and 117 increased on both (the second and third) days after the invasion of the phytopathogen (by 50% each day) versus line 112, where the increase (by 50%) was observable only on day 2 and then remained the same.

**Figure 6:**
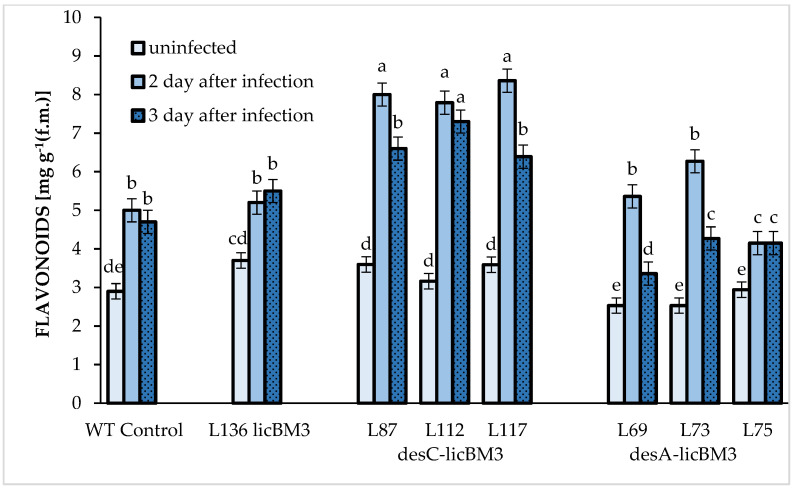
Changes in the number of flavonoids in the leaves of control plants of potato cultivar Skoroplodnyi and transgenic lines, expressing gene *licB*M3, gene *desC-licB*M3 (*desC* plants) and gene *desA-licB*M3 (*desA* plants) after infection with *P. infestans* isolate 1.2.3.4.5.6.7.8.9.10.11 XYZ. Means ± SDs, *n* = 3. Different Latin letters above the bars denote statistically significant differences at *p* *≤* 0.05.

Flavonoids are the main components in the secondary metabolites of leaves of potato plants (Figure 5 and Figure 6). During the infection with *P. infestans*, the number of flavonoids was raised (Figure 6).

Their content was increased on the second day after inoculation in the control plants and the plants of the transgenic *licB*M3 line and further remained unchanged. As for the transgenic *desC* plants, the changes in the accumulation of flavonoids after infection were the most pronounced as compared with the other variants. In particular, their content in line 112 exceeded 2.5-fold that of the uninfected leaves as early as day 2 of infection and later remained at the same level. In lines 87 and 117, the flavonoid content in the leaves infected with *P. infestans* increased on day 2 after inoculation (more than two-fold) but somewhat decreased (by 20%) on day 3. The transgenic *desA* plants also displayed increased flavonoid content as compared with the uninfected plants. In particular, this content doubled in lines 69 and 73 and increased by 40% in line 75 on the second day after infection as compared with control potato plants. On the third day, the flavonoid content either decreased (by 50% in lines 69 and 73) or remained the same (line 75).

## 3. Discussion

### 3.1. Morphophysiological Characteristics of Potato Plants

The morphometric parameters of plants depend on the growing conditions and the action of exogenous factors and are important in assessing their physiological state [25,26]. Potato plants of the cv. Skoroplodnyi expressing the *desA* gene showed the highest values in height, weight, number of internodes and water content in leaves in comparison with all other variants (Table 1). It can be concluded that they significantly differed from the control non-transgenic potato plants and transgenic *desC* plants. The mass of all plant organs depends on the water content in them, which is an important indicator in assessing the physiological state of plants [27]. It had similar and statistically equal values in the leaves of potato plants of all studied variants.

Water status regulation of plant leaves depends on the presence of stomata and their functional activity [28,29]. They provide the main pathway for the exchange of CO_2_, O_2_ and water between the atmosphere and plant tissues. Research on stomata localization on leaf surfaces in potato plants expressing desaturase genes showed a decrease in their number, especially on adaxial surfaces.

### 3.2. Leaf Resistance in Control and Transgenic Potato Plants to Phytophthora infestans

The phytopathogen *P. infestans* has a severe and damaging effect on the organs and tissues of plants [30]. Its impact depends on the interaction between the pathogen and the host, race-specific pathogen resistance, penetration by the pathogen and the defense system of plant cells [31].

In this study, the transgenic potato plants (cv. Skoroplodnyi) expressing the heterologous genes *desC*-*licB*M3 and *desA*-*licB*M3 displayed an increased resistance to infection by a complex race of *P. infestans* as compared with untransformed plants or transgenic plants expressing the reporter gene *licB*M3 (Table 3, Figure 3). Expression of genes encoding acyl-lipid desaturases with different substrate specificities in potato plants enhanced their resistance to *P. infestans* almost equally. A similar pattern was observed when this phytopathogen infected transgenic *S. tuberosum* L. (cv. Desnitsa) plants expressing the *desA* gene from *Synechocystis* [24].

### 3.3. Lipid Peroxidation in Control and Transgenic Potato Plants under Normal Conditions and during Phytophthora infestation

One of the criteria in assessing plant resistance to stress impacts is the level of LPO [32,33,34]. MDA is formed at all stages of plant growth since the cells (especially photosynthesizing ones) actively produce reactive oxygen species (ROS) in chloroplasts, mitochondria, peroxisomes and apoplasts [35,36]. The number of ROS depends on the relative rates of their production and destruction, lifespan of their main forms and acting factors.

Our results suggest that the lipid peroxidation (LPO) level in the leaves of the in vivo-grown transgenic potato plants expressing desaturase genes was higher as compared with the corresponding control plants (Figure 4). The malondialdehyde (MDA) content in the *desC* plants was two-fold higher than that in control plants and even three-fold higher than that in the *desA* plants. These results allow us to assume that the expression of heterologous desaturase genes in potato plants is accompanied by an increase in peroxidation of polyunsaturated fatty acids and, consequently, enhanced MDA accumulation, which was more pronounced in the *desA* plants. It also cannot be excluded that the functioning of the antioxidant system shifted towards excess ROS accumulation, thereby initiating LPO.

The infection with a potato-late-blight agent caused an increase in the MDA content in the leaves of control plants and the transgenic *desA* plants on the third day after inoculation. However, the MDA level was unaffected in *desC* plants compared with control and *desA* plants. The results suggest that *P. infestans* infection of the potato plants expressing the heterologous gene *desC-licBM3* failed to change the antioxidant status of these plants. There was an absence of changes in the LPO level in the transgenic potato plants cv. Desnitsa expressing *desA-licBM3* [24] after a hypothermic impact.

### 3.4. Phenolic Compounds in Potato Leaf Resistance to Phytophthora infestans

The antioxidant system comprising high- and low-molecular-weight antioxidants plays an important role in the regulation of the ROS and LPO levels [5,6,8]. Phenolic compounds belong to low-molecular-weight antioxidants and are among the most abundant plant secondary metabolites. The main classes of such compounds are represented by phenylpropanoids, flavonoids and phenolic polymers, in particular, lignin [7,9,37]. Because of their chemical properties, they readily interact with ROS, thereby protecting cells from their negative effects [38,39].

The involvement of phenolic compounds in plant adaptation to different stress effects has been repeatedly discussed [7,9,40]. It is known that they prevent penetration of many pathogenic microorganisms and inhibit their growth and development and production of the cell-toxic metabolites. In addition, they decrease the intensity of oxidative stress [5,7,11,41]. Some phenolic compounds synthesized in plants have fungicide activity. For example, caffeic acid, accumulated in potato tubers, protects them from *Phthorimaea operculella* [42]. Plants respond to the penetration of pathogens by an increase in the production of different phenolic compounds, which is seen as the host’s resistance to the pathogen [12].

In our study, a determination of the total phenolic compound content in potato plant leaves has shown that the control plants and the transgenic *desA* plants have almost equal levels of these compounds, while their level in transgenic *desC* plants was 50% higher (Figure 5). The number of total phenolic compounds increased in all studied variants after *P. infestans* infection. This increase was observed on the second day after infection in the control plants and *desA* plants and further remained at the same level. As for the *desC* plants, an increase in the total phenolic compound content was observed in most cases not only on the second day after *P. infestans* infection but on the third day as well (lines 87 and 117 versus line 112). Despite some differences in the phenolic compound accumulation pattern during the infection period, all transgenic plants expressing the *desC-licB*M3 gene displayed approximately the same level of these metabolites on the third day, and this level considerably exceeded that of all remaining variants. Thus, our results suggest that the infection of plants with the complex race of *P. infestans* enhances the rapid activation of phenolic compounds’ biosynthesis in the leaves, thereby leading to the most pronounced changes in their accumulation as early as the second day of the interaction between the pathogen and the host. An increase in the total content of phenolic compounds during infection with phytopathogens was also observed in other plant species. In particular, an increase in accumulation of the phenolic compounds with high antioxidant activity is determined by the protective response of the host birch (*Betula pendula*) to stem rot diseases [43].

As it was mentioned above, flavonoids are among the most abundant compounds of a phenolic nature that are characteristic of green plant tissues [7,9,44]. Their antipathogenic properties have been repeatedly demonstrated, which may result from both their non-specific effect and antioxidant activity [11,13,45]. Flavonoids are known to inhibit ROS, which are generated by both pathogenic microorganisms and plants as a result of infection [40,46]. Flavonoid content has been shown to correlate with plant resistance to fungal diseases and some pests; growth inhibition of fungal mycelium is regarded as a defense reaction [13].

The flavonoid content determined in the plant leaves of all studied potato lines not infected with *P. infestans* was almost the same (Figure 6). However, this content increased as early as the second day after infection. The number of flavonoids in the transgenic *desC* potato lines was 43% higher as compared with the plants of transgenic *desA* lines and 65% higher as compared with the control plants. Afterwards (day 3 of infection), this content did not change in the control variants or in two transgenic plant lines expressing the *desC-licB*M3 gene (line 112) and *desA-licB*M3 gene (line 75). As for the remaining lines, the number of flavonoids remained at the same level as on the second day of *P. infestans* infection.

A comparison of the data on the total number of phenolic compounds and flavonoid contents in potato plant leaves demonstrates that infection with the phytopathogen induces a rapid activation of flavonoid accumulation, which is more pronounced in transgenic *desC* plants. These changes can be regarded as the earliest defense mechanism used by infected plants to prevent cell death [13]. As for the regulation of phenolic compounds production in potato plant leaves at later stages of infection, the biosynthesis of these secondary metabolites did not change in the control variants versus the transgenic lines with introduced desaturase genes. Accumulation of flavonoids in most cases decreased under preservation of the total phenolic compound content in these lines.

## 4. Materials and Methods

### 4.1. Generation of Transgenic Plants

Transgenic potato plants (*Solanum tuberosum* L.) of the early-maturing cultivar Skoroplodnyi from the “Bank of virus-free potato cultivars” of the Russian Potato Research Center, Kraskovo, Russia, were obtained by agrobacterial transformation [47,48,49]. Cells of *Agrobacterium tumefaciens* AGL0 strain comprised the genetic constructs based on expression vector pBI. In addition to the marker cassette with the NPTII gene conferring the resistance to kanamycin, three variants of cassettes of interest were in the expression vector (Supplementary Materials, Figure S1). The first expression cassette played the role of a control in transformation experiments and contained only the lichenase gene (*licB*M3). The second expression cassette contained hybrid gene of ∆12-acyl-lipid desaturase from the cyanobacterium *Synechocystis* sp. PCC6803 fused in frame with marker gene of bacterial termostable lichenase (*desC-licB*M3). The third cassette contained hybrid gene of ∆9-acyl-lipid desaturase from the cyanobacterium *Synechococcus vulcanus* fused in frame with marker gene of bacterial termostable lichenase (*desC-licB*M3). All the genes of interest were under the control of a strong constitutive CaMV 35S promoter (Supplementary Materials, Figure S1) [48,49,50,51].

The expression of the gene of interest was confirmed by the results of the polymerase chain reaction, and the level of expression was assessed by the activity of lichenase, which is the moiety of the fusion protein.

### 4.2. Molecular Confirmation of Transgenic Plants

Molecular confirmation was performed by multiplex polymerase chain reaction (PCR). Primers, their ratio and PCR conditions have been described previously [16]. The previously isolated total DNA of plants was used as a template. DNA electrophoretic separation was performed in 1% agarose gel prepared in TAE buffer (Supplementary Materials, Figure S2).

### 4.3. RNA Analysis

To analyze the expression of *desA, desC* and *licB*, leaves of 60-day-old potato plants were collected for analysis. Total RNA was extracted from these samples using an RNAzol kit (SibEnzyme Ltd., Novosibirsk, Russia) according to the manufacturer’s instructions. The RNA concentration and quality were confirmed by a NanoDrop spectrophotometer (NanoDrop ND-2000 spectrophotometer (NanoDrop Technologies, Wilmington, DE, USA). Samples were prepared by the standard method using a set of reagents for real-time PCR in the presence of Sybr Green (Syntol). Quantitative real-time PCR reaction was carried out using the CFX 96 Real-Time System thermal cycler (Bio-Rad, Hercules, CA, USA). (SYBR) Green reagent in a LightCycler 480 Real-Time PCR System (Roche Diagnostics, Basel, Switzerland). The GaPDh gene encoding the glyceraldehyde-3-phosphate dehydrogenase protein was taken as the reference gene. Relative expression was determined through a previously described method [52] (Supplementary Materials, Figures S3 and S4).

### 4.4. Experimental Conditions

Leaf explants of previously in vitro-micropropagated potato plants were used for transformation. The kanamycin-resistant regenerants were chosen for the experiment. More than 25 lines were created with each construct (data not shown and partially described earlier). Then, the plants that did not have significant phenotypic differences from the initial cultivar were subjected to cold stress (–6 °C) for one day. Lines were selected that demonstrated the restoration of turgor on the next day after returning to normal cultivation conditions (data not shown). Transgenic *desC-licBM*3 lines 87, 112 and 117; transgenic *desA-licBM3* lines 69, 73 and 75; and a transgenic *licBM3* line, which is a transgenic control, were selected for further analysis [47,48,49] (Supplementary Materials). Plants of initial cultivar Skoroplodnyi were used as a non-transgenic control.

The aseptic plants were planted into pots (V = 1 L) with growth substrate (soil Vozdushnyi, BIUD, Russia) and cultivated for 60 days in a climate chamber (Institute of Plant Physiology RAS) for a 16 h photoperiod (illumination, 1000 lx) at 75% humidity.

**Figure 7:**
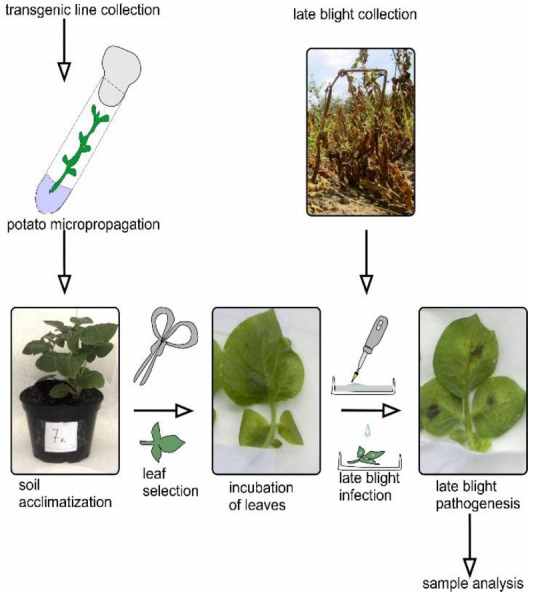
Scheme of inoculation of potato plants with infection with isolate 1.2.3.4.5.6.7.8.9.10.11 XYZ of *P. infestans*.

Each pot contained 1 plant, and three replicates were made for each line. The general scheme of the experiment is represented in Figure 7.

### 4.5. Evaluation of Morphometric Parameters in Potato Plants

The assessment of the morphophysiological state of plants was carried out using traditional methods for plant physiology. The height and number of internodes in plants of all variants were determined [50].

The weight of each plant and leaves separated from it was analyzed by weighing them on an analytical balance.

The water content was determined after drying the plant leaves to constant weight at 70 °C [51].

### 4.6. Stomata Distribution on the Leaf Surface

Microscope slides were used for microscopic examination of adaxial and abaxial surface of potato plant leaves. After cutting, the epidermal layer was carefully separated from the leaf and placed on a microscope slide [53] with a cover slip over the sample and viewed under the compound light microscope according to the procedure [54]. Stomata were counted on micrographs of five to seven 0.66 mm^2^ areas from each replicate leaf [55].

### 4.7. Inoculation of Potato Leaves with Phytophthora infestans and Assessment of Their Resistance

The leaves of control and transgenic potato plants were infected with the pathogen, and the degree of their resistance was assessed according to techniques which were previously described [24].

A complex race of the fungus *P. infestans* 1.2.3.4.5.6.7.8.9.10.11 XYZ, carrying 13 virulence genes, was obtained from pathogen culture collections in Russian Potato Research Center and maintained on oatmeal agar slants for 14 days at 18–19 °C [56]. The suspension for inoculation was produced by washing off conidia with sterile water from the nutrient medium with pathogen mycelium. Concentration of the suspension was 20–25 conidia per microscopic field (magnification, ×120) or 30 × 10^3^ mL^−1^.

Three to five leaves from the middle part of a plant were used for inoculation by applying 100 µL of the suspension of conidia onto the underside of each leaf lobe. The inoculated leaves were placed into a cuvette on wet filter paper with their lower part facing upwards and incubated in a diffuse light for 3 days at +18 °C.

The degree of infection of plant leaves was estimated using the following scale: 9 points, very high resistance (no signs of affection); 7 points, high resistance (25% of leaf surface area affected); 5 points, medium resistance (25 to 50% of leaf surface area affected); 3 points, low resistance (50–75% of leaf surface area affected); and 1 point, susceptible (over 75% of leaf surface area affected) [24].

Fragments of control and transgenic potato plant leaves adjacent to the area affected by the phytopathogen were used for biochemical assays.

### 4.8. Determining the Content of Malondialdehyde (MDA)

The MDA content was determined in the reaction with thiobarbituric acid (TBA). Plant material was homogenized in 0.1 M Tris–HCl buffer (pH 7.5) containing 0.35 M NaCl and supplemented with 0.5% TBA solution in 20% aqueous solution of trichloroacetic acid [57]. The reaction mixture was incubated in a boiling water bath for 30 min and cooled to measure the optical density in a spectrophotometer at 532 nm. The MDA concentration was calculated using the standard equation with a molar extinction coefficient (1.56 × 10^–5^ cm^−1^ M^−1^).

### 4.9. Determining the Content of Total Phenolic Compounds and Flavonoids

Phenolic compounds were extracted from plant material with 96% ethanol at 45 °C for 45 min [58]. The homogenates were centrifuged (2 min, 13,260× *g*); the supernatant was collected and assayed by spectrophotometry to determine the total content of phenolic compounds with the Folin–Ciocalteu reagent (725 nm) and the content of flavonoids with 1% aluminum chloride (415 nm) [59,60]. In both cases, the calibration curves were constructed according to rutin and their content was expressed as mg rutin equivalent/g fresh mass.

### 4.10. Obtaining Protein Extracts from Plants

The plant material was triturated in liquid nitrogen; 1 mL of 50 mM Tris–HCl buffer (pH 8.0) was added and incubated on ice for an hour. It was centrifuged at 16,000 rpm for 20 min, and the supernatant was used as a protein lysate for further work.

### 4.11. Determining the Molecular Weight of Fusion Proteins by Enzymogram Method

Enzymograms were obtained by gel staining after separation of protein preparations in PAGE containing 0.1% lichenan. Electrophoresis was performed in 12% PAGE in the presence of sodium dodecyl sulfate (SDS). Plant protein preparations (0.1–100 μg) were applied to the gel after pre-heating in a buffer for sample application (0.312 M Tris–HCl, pH 6.8; 25% β-mercaptoethanol; 10% SDS; 50% glycerol; 375 mg/mL of bromophenol blue) at 100 °C (boiling water bath) for 10 min. After the end of electrophoresis, the gels were washed in 50 mM Tris–HCl (pH 8.0) at room temperature for 30 min. Then, the gels were incubated at 65 °C (1–3 h). The enzyme lichenase activity was determined by staining the gels with a 0.5% Congo Red solution (Sigma-Aldrich, St. Louis, MO, USA) followed by washing in 1 M sodium chloride. In this case, a clear area was found in place of the active protein, since the dye binds only to non-hydrolyzed lichenan.

### 4.12. Statistical Analysis

All variants of the described experiments and assays were performed in triplicate. SigmaPlot 12.3 (http://www.sigmaplot.co.uk accessed on 9 February 2021) and Microsoft Excel were used for statistical processing. The tables and plots show arithmetic means (M) and their standard errors (±SEM). Superscripts show statistical significance of the differences between means according to Tukey’s test at *p* *≤* 0.05. 

## 5. Conclusions

Thus, an expression of the heterologous genes *desC*-*licB*M3 and *desA*-*licB*M3 in potato cultivar Skoroplodnyi plants was accompanied by changes in the phenolics accumulation and LPO level. This was more pronounced in the transgenic *desC* plants. These data suggest that the changes in physical properties of the membranes in transgenic plants (at the expense of a higher content of unsaturated fatty acids) can modulate the conformation and/or activity of the protein complexes integrated into membrane lipids. Most of them are either receptors or sensors and are involved in signal transduction in the case of stress impacts. This can lead to considerable changes in physiological and biochemical characteristics of transgenic plants, including accumulation of phenolic compounds, low-molecular-weight antioxidants involved in the plant tissue defense against various stress factors, including phytopathogens.

The findings of this study indicate the important role of desaturases in the regulation of potato plants’ resistance to *P. infestans* infection. In the future, the results can be used to develop new biotechnological approaches.

## 6. Patents

The method for obtaining transgenic potato plants is outlined in https://www.freepatent.ru/patents/2505955 (accessed on 9 February 2021).

## Figures and Tables

**Table 1:** Morphophysiological characteristics of the control and transgenic plants of potato cultivar Skoroplodnyi (plant age, 60 days).

	Variants	WT Plants (Control)	*licB*M3 Plants, L136	*desC* Plants			*desA* Plants		
Parameter				L87	L112	L117	L69	L73	L75
Plant height *, cm		17.3 ± 1.8 ^C^	20.3 ± 1.3 ^B^	19.4 ± 1.1 ^B^	17.1 ± 0.9 ^C^	21.4 ± 1.4 ^B^	25.7 ± 2.1 ^A^	24.3 ± 2.3 ^A^	25.2 ± 2.14 ^A^
Number of internodes *, pcs		9 ± 1 ^B^	10 ± 1 ^B^	10 ± 1 ^B^	10 ± 1 ^B^	10 ± 1 ^B^	15 ± 1 ^A^	15 ± 1 ^A^	15 ± 1 ^A^
Plant weight *, g		3.21 ± 0.21 ^B^	3.69 ± 0.21 ^B^	3.56 ± 0.2 ^B^	3.89 ± 0.23 ^B^	3.82 ± 0.26 ^B^	6.42 ± 0.39 ^A^	6.35 ± 0.42 ^A^	6.45 ± 0.38 ^A^
Leaf weight *, g		2.56 ± 0.15 ^B^	3.02 ± 0.16 ^A^	3.05 ± 0.1 ^A^	3.11 ± 0.15 ^A^	3.01 ± 0.17 ^A^	3.46 ± 0.13 ^A^	3.31 ± 0.12 ^A^	3.23 ± 1.11 ^A^
Water content in leaves, %		75.3 ± 2.1 ^A^	81.6 ± 2.3 ^A^	85.0 ± 2.2 ^A^	83.4 ± 1.9 ^A^	87.0 ± 2.1 ^A^	90.9 ± 2.4 ^A^	92.1 ± 2.4 ^A^	92.6 ± 2.5 ^A^

* calculation is produced per plant. Abbreviations: WT— control plants; *licB*M3 plants (line 136) —transgenic potato plants expressing the reporter gene of thermostable lichenase (*licB*M3); *desC* plants (L87, L112, L117)—transgenic potato plants expressing the hybrid gene of ∆9-acyl-lipid desaturase from the cyanobacterium *S. vulcanus* translationally fused with the sequence of reporter gene *licB*M3; *desA* plants (L69, L73, L75)—transgenic potato plants expressing the hybrid gene of ∆12-acyl-lipid desaturase from the cyanobacterium *Synechocystis* sp. PCC6803 translationally fused with the sequence of reporter gene *licB*M3. Means ± SDs, *n* = 5. Different letters above the bars denote statistically significant differences at *p* *≤* 0.05.

**Table 2:** Epidermis structure parameters on the abaxial and adaxial leaf surfaces of control potato plants of the cultivar Skoroplodnyi and transgenic lines, expressing the genes *licB*M3, *desC-licB*M3 or *desA-licB*M3 (plant age, 60 days).

Variants	Number of Stomata		Area Stomata, mm		Area Epidermis Cells		Stomatal Index	
	Adaxial	Abaxial	Adaxial	Abaxial	Adaxial	Abaxial	Adaxial	Abaxial
WT (control)	1	23	39	40.2	79.4	140.8	2.07	47.8
*licB*M3 plants (L136)	4	16	52.8	47.9	84.4	88.2	8.3	33.3
*desC* plants (L87)	1	10	43.1	31.3	127.3	118.9	2.07	20.7
*desA* plants (L69)	2	9	44.5	48.1	160.6	93.3	4.15	18.7

Variants: WT —control plants; *licB*M3 plants (line 136)—transgenic potato plants expressing the reporter gene of thermostable lichenase (*licB*M3); *desC* plants (L87, L112, L117)—transgenic potato plants expressing the hybrid genes of ∆9-acyl-lipid desaturase from the cyanobacterium *S. vulcanus* translationally fused with the sequence of reporter gene *licB*M3; *desA* plants (L69, L73, L75)—transgenic potato plants expressing the hybrid gene of ∆12-acyl-lipid desaturase from the cyanobacterium *Synechocystis* sp. PCC6803 translationally fused with the sequence of reporter gene *licB*M3.

**Table 3:** Resistance indicator of control and the transgenic plants of potato cultivar Skoroplodnyi expressing gene *licB*M3, gene *desC-licB*M3 and gene *desA-licB*M3 after infection with the complex race of *P. infestans*.

Transgene	Line	Resistance Indicator, Points	
		Second Day after Infection	Third Day after Infection
	WT (control)	5.5 ± 0.7 ^E^	4.2 ± 0.41 ^D^
*licB*M3	L136	6.2 ± 0.2 ^D^	4.5 ± 0.6 ^D^
*desC*-*licB*M3	L87	7.1 ± 0.38 ^C^	6.8 ± 0.88 ^B^
	L112	6.9 ± 0.27 ^C^	6.7 ± 0.2 ^B^
	L117	7.3 ± 0.1 ^C^	6.2 ± 0.54 ^C^
*desA*-*lic*BM3	L69	7.8 ± 0.68 ^B^	7 ± 0.82 ^B^
	L73	8.1 ± 0.55 ^AB^	7.5 ± 0.43 ^A^
	L75	8.4 ± 0.61 ^A^	7.2 ± 0.17 ^AB^

WT (cv. Skoroplodnyi)—control plants; *licB*M3—transgenic plants expressing the reporter gene of thermostable lichenase (*licB*M3); *desC-licB*M3—transgenic plants expressing the hybrid gene of ∆9-acyl-lipid desaturase from *Synechococcus vulcanus* translationally fused with the sequence of reporter gene *licB*M3 (lines 87, 112, 117); and *desA-licB*M3—transgenic plants expressing the hybrid gene of ∆12-acyl-lipid desaturase from *Synechocystis* sp. PCC6803 translationally fused with the sequence of reporter gene *licB*M3. (lines 69, 73, 75). Results are expressed as mean ± standard deviations, *n* = 3. Different Latin letters above the numbers denote statistically significant differences at *p* *≤* 0.05.

## Data Availability

Data are contained within the article and Supplementary Materials.

## Supplementary Materials

The following are available online at https://www.mdpi.com/article/10.3390/plants11030288/s1.
