# Supplementary material for: Response of Transgenic Potato Plants Expressing Heterologous Genes of ∆9- or ∆12-Acyl-lipid Desaturases to Phytophthora infestans Infection
Source: Plants (Basel). 2022 Jan 21;11(3):288. doi: 10.3390/plants11030288 (PMC8840463; doi:10.3390/plants11030288)
Supplement: Supplementary file 1 [file plants-11-00288-s001.zip › plants-1501502-supplementary.pdf]

Supplementary Material (**Figures S1–S5**)

Tsypurskaya, E.V.; Nikolaeva, T.N.; Lapshin, P.V.; Nechaeva, T.L.; Yuorieva, N.O.; Baranova, E.N.; Derevyagina, M.K.; Nazarenko, L.V.; Goldenkova-Pavlova, I.V.; Zagoskina, N.V. Response of Transgenic Potato Plants Expressing Heterologous Genes of Δ9- or Δ12-acyl-lipid Desaturases to *Phytophthora infestans* infection. *Plants* **2022**, *11*

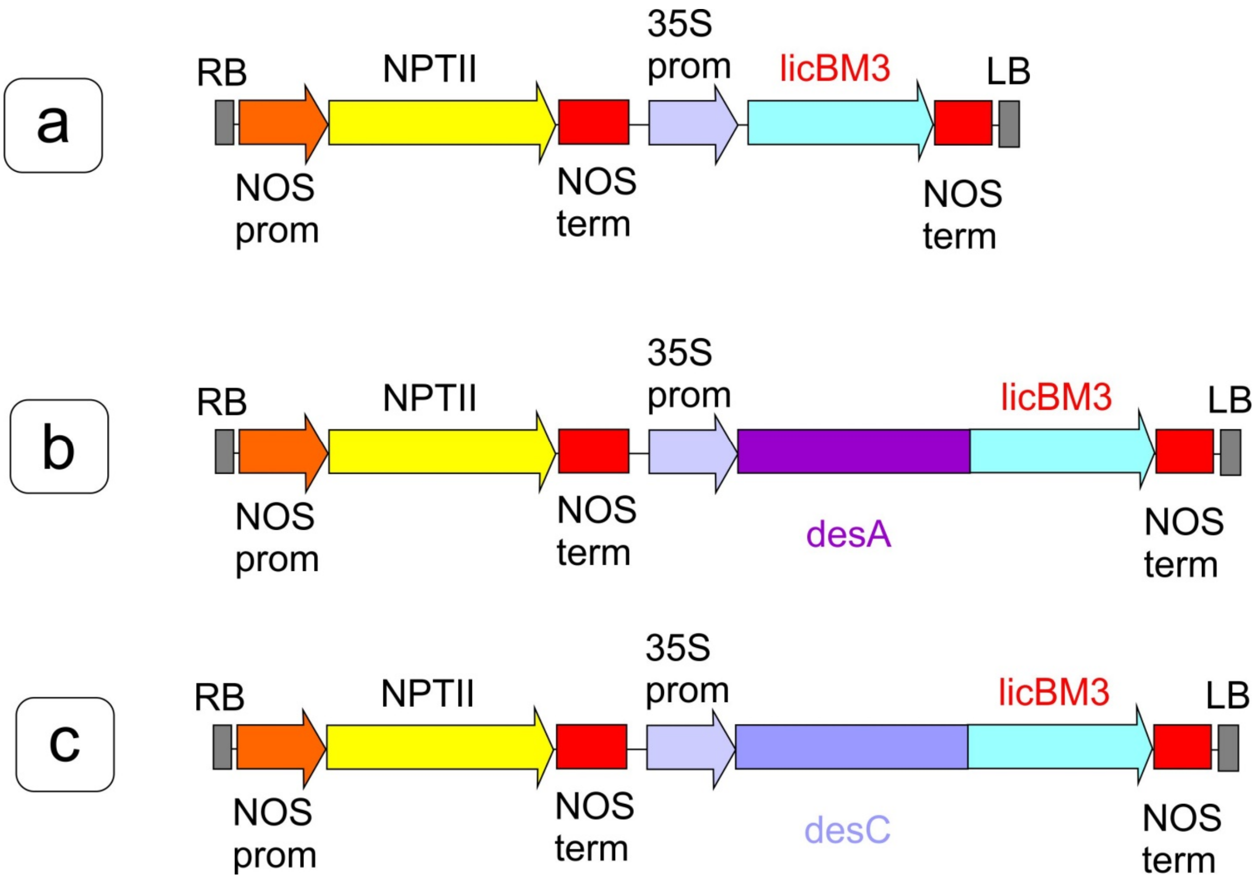

Figure S1. Scheme of T-DNA region of plant expression vectors for plant transformation harboring the reporter *licBM3* gene (**a**); hybrid *desA-licBM3* gene (**b**); and hybrid *desC-licBM3* gene (**c**). Abbreviations: RB – right border; LB – left border; NOSprom - promoter of the nptII gene; NPTII – the marker gene encoding neomycin transferase II; NOSterm – terminator of nopaline synthase gene; 35Sprom – constitutive CaMV 35S promoter for expression of the gene of interest; *desA*—the gene encoding Δ12 acyl-lipid desaturase; *desC*—the gene encoding Δ9 acyl-lipid desaturase; *licBM3*—sequence of the reporter gene encoding thermostable β-1,3-1,4-glucanase (lichenase) of *Clostridium thermocellum.*

The presence of sequences for target heterologous gene in the genome of the selected plant transformants was confirmed by multiplex PCR, like previously described [16]. Regenerated plants resistant to selective agents were analyzed by multiplex PCR, which allows detecting the presence of the desA, licBM3 and nos terminator (by the presence of amplicons of 949, 642, and 128 bp, respectively). DNA quality was assessed by amplification of the actin gene fragment (432 bp). The absence of the virD1 gene amplicon (351 bp) indicated the complete elimination of Agrobacteria after genetic transformation (Supplementary Materials Figure S2). Expression of the desA-licBM3, desC-licBM3 hybrid gene and licB gene as a control at the level of RNA product formation was confirmed by the method RT-PCR (we use same primers for cDNA detection)[16] (Supplementary Materials Figure S3). Expression of the desA-licBM3, desC-licBM3 hybrid gene and licB gene for tree different plants were demonstrate on Supplementary Materials Figure S4).

Also expression of the desC-licBM3 hybrid gene at the level of protein product formation was confirmed by the method of enzymograms. Briefly, electrophoresis was performed in 12% PAGE in the presence of Ds-Na. Lichenan (0.1%) was added to the separation gel prior to polymerization. Plant extracts (0.1–20 μg of total protein) were applied to the gel after preliminary heating in the buffer for applying samples for 8 min in a boiling water bath. The clarified stripes on zymograms indicate that the synthesis of protein products with a molecular weight corresponding to the theor etically calculated one occurs in plants: about 56 kDa for a hybrid protein. (Supplemental Fig. S5).

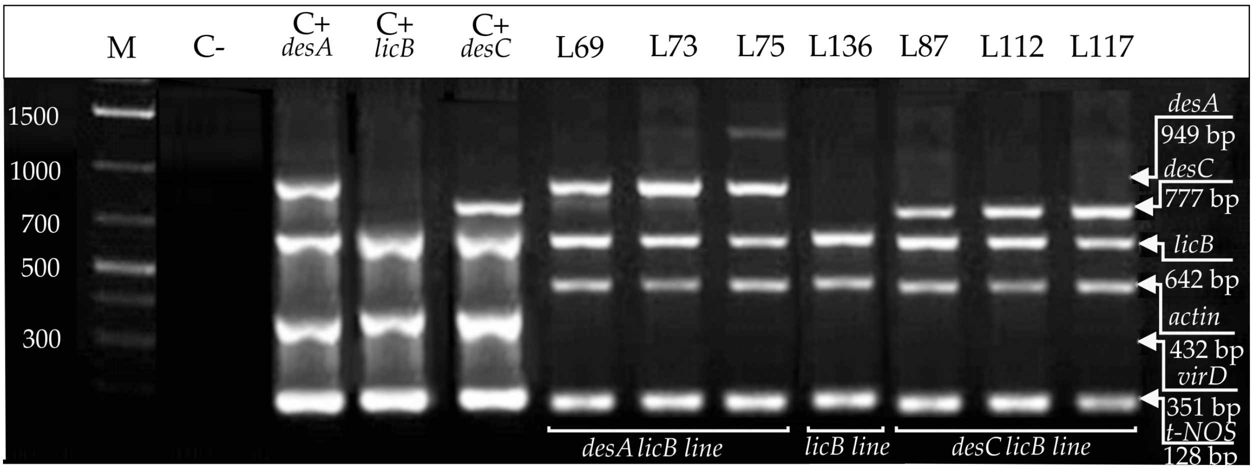

Figure S2. Relative expression levels of genes of *desA, desC* and *licB* in potato transgenic line. R1,R2,R3 - Show the expression level determined by RT-PCR.

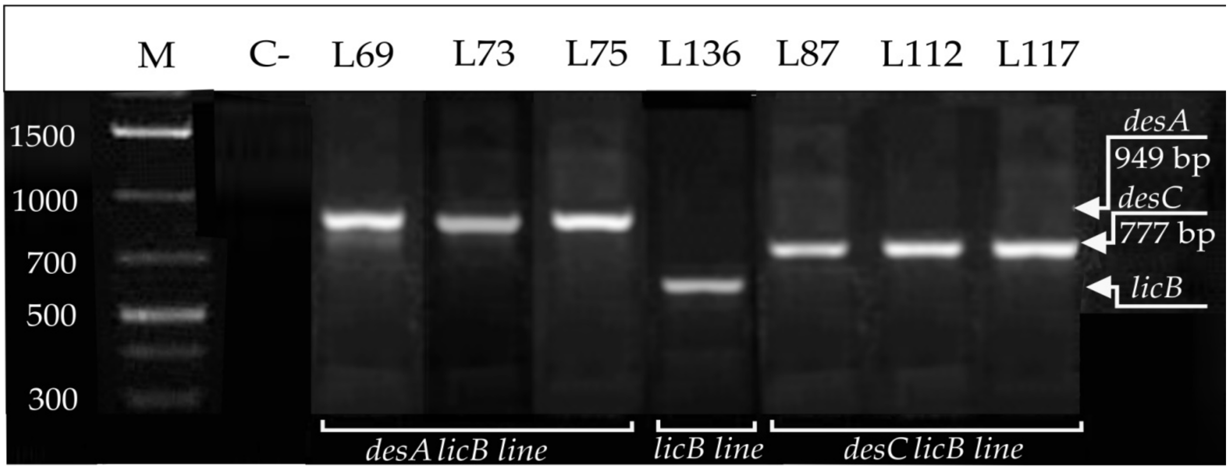

Figure S3. RT-PCR analysis of the expression level of *desA*, *decC* and *licB* genes in transgenic potato plants using the EF-1α gene, corresponding to actin, as a control. M –marker; C- –WT; desAlicB – L69,L73, L75; licB – L136 ; desClicB – L87,L112, L117.

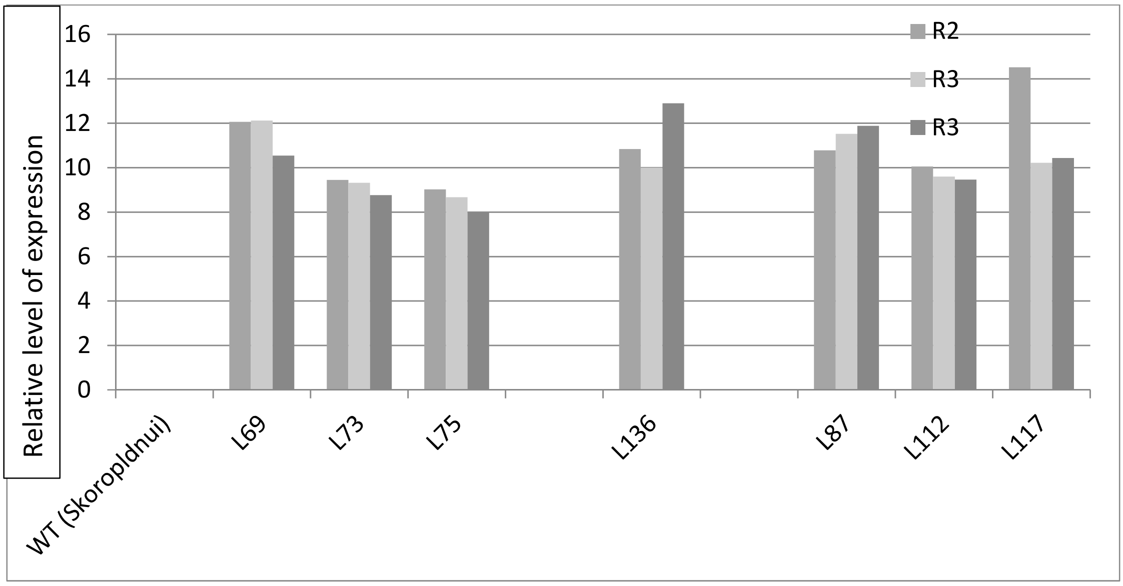

Figure S4. Relative expression levels of genes of *desA, desC* and *licB* in potato transgenic line. R1,R2,R3 - Show the expression level determined by RT-PCR.

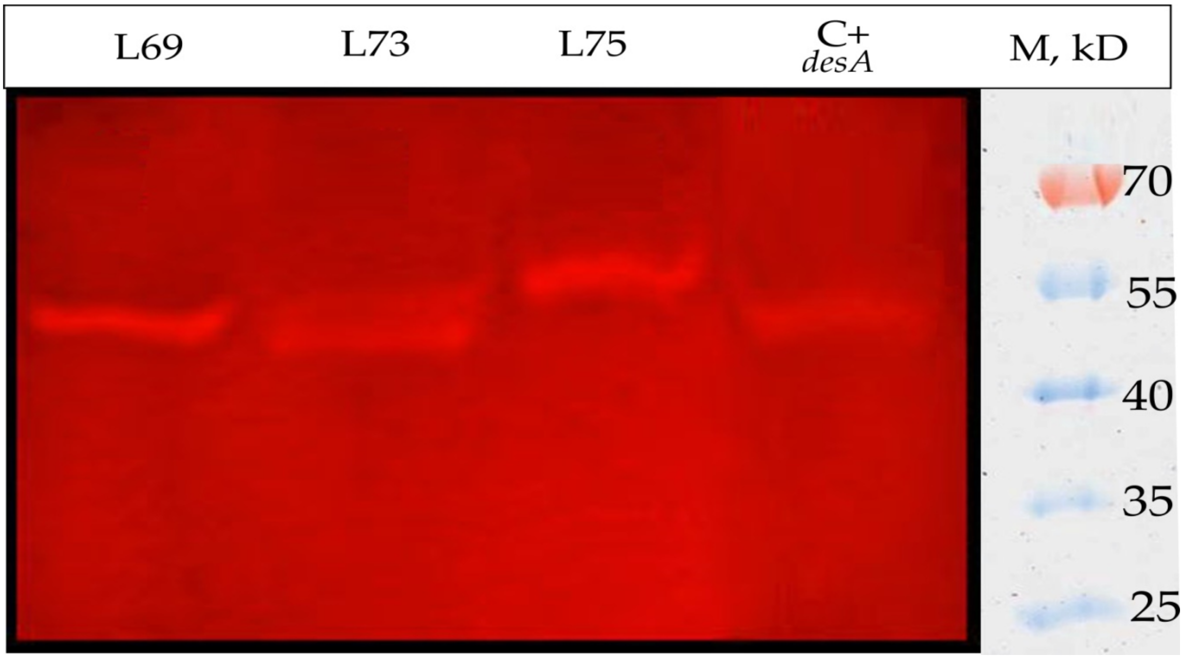

Figure S5. Expression of the desC-licBM3 hybrid gene at the level of protein product formation was confirmed by the enzymograms method .
